# Cytocidal Effect of Irradiation on Gastric Cancer Cells Infected with a Recombinant Mammalian Orthoreovirus Expressing a Membrane-Targeted KillerRed

**DOI:** 10.3390/ph17010079

**Published:** 2024-01-08

**Authors:** Yoshinori Shirasaka, Kentaro Yamada, Tsuyoshi Etoh, Kazuko Noguchi, Takumi Hasegawa, Katsuhiro Ogawa, Takeshi Kobayashi, Akira Nishizono, Masafumi Inomata

**Affiliations:** 1 Department of Gastroenterological and Pediatric Surgery, Oita University Faculty of Medicine, 1-1 Idaigaoka, Hasamamachi, Yufu City 879-5593, Oita, Japan; yshirasaka@oita-u.ac.jp (Y.S.); t-hasegawa@oita-u.ac.jp (T.H.); katsu-ogawa@oita-u.ac.jp (K.O.); inomata@oita-u.ac.jp (M.I.); 2 Laboratory of Veterinary Public Health, Department of Veterinary Sciences, Faculty of Agriculture, University of Miyazaki, 1-1 Gakuenkibanadai-Nishi, Miyazaki City 889-2192, Miyazaki, Japan; iikaz-n@cc.miyazaki-u.ac.jp; 3 Department of Microbiology, Oita University Faculty of Medicine, 1-1 Idaigaoka, Hasamamachi, Yufu City 879-5593, Oita, Japan; a24zono@oita-u.ac.jp; 4 Research Center for GLOBAL and LOCAL Infectious Diseases, Oita University, 1-1 Idaigaoka, Hasamamachi, Yufu City 879-5593, Oita, Japan; 5 Department of Virology, Research Institute for Microbial Diseases, Osaka University, 3-1 Yamadaoka, Suita City 565-0871, Osaka, Japan; tkobayashi@biken.osaka-u.ac.jp

**Keywords:** oncolytic reovirus, reverse genetics, fluorescent imaging, KillerRed, gastric cancer

## Abstract

The outcomes of unresectable gastric cancer (GC) are unfavorable even with chemotherapy; therefore, a new treatment modality is required. The combination of an oncolytic virus and photodynamic therapy can be one of the promising modalities to overcome this. Mammalian orthoreovirus (MRV) is an oncolytic virus that has been used in clinical trials for several cancers. In this study, we developed and evaluated a recombinant MRV strain type 3 Dearing (T3D) that expresses membrane-targeting KillerRed (KRmem), a phototoxic fluorescent protein that produces cytotoxic reactive oxygen species upon light irradiation. KRmem was fused in-frame to the 3′ end of the σ2 viral gene in the S2 segment using a 2A peptide linker, enabling the expression of multiple proteins from a single transcript. RNA electrophoresis, Western blotting, and immunofluorescence analyses confirmed functional insertion of KRmem into the recombinant virus. The growth activity of the recombinant virus was comparable to that of the wild-type MRV in a cultured cell line. The recombinant virus infected two GC cell lines (MKN45P and MKN7), and a significant cytocidal effect was observed in MKN45P cells infected with the recombinant virus after light irradiation. Thus, recombinant MRV-expressing KRmem has the potential to serve as a novel treatment tool for GC.

## 1. Introduction

Gastric cancer (GC) is one of the most common causes of cancer-related death worldwide. The prognosis of patients with resectable GCs can improve after surgery, and chemotherapy is considered the standard treatment for unresectable and recurrent GCs. The median survival time for chemotherapy alone has been reported to be approximately 13 months [1,2], the outcomes of which remain unsatisfactory since radiation therapy is insufficient for the treatment of GC. Radiation therapy is sometimes administered simultaneously with chemotherapy, but only as an auxiliary treatment for pain control from bone metastases or bleeding from lymph node metastases [3]. Therefore, it is necessary to develop new therapeutic tools to improve the prognosis of patients with GC.

Photodynamic therapy (PDT) is a minimally invasive treatment with tumor-killing effects induced by photosensitizer injection and light irradiation [4,5]. Photosensitizers produce reactive oxygen species (ROS), which exhibit cytocidal effects when irradiated with light of a specific wavelength. Photosensitizers (such as porfimer sodium, talaporfin, and 5-aminolevulinic acid) are currently covered by national insurance in Japan and are used for the treatment of malignant neoplasms (including esophageal, lung, and cervical cancers) [6,7,8]. However, these photosensitizers can also accumulate in normal tissues, resulting in the potential risk of skin damage due to photosensitivity, as well as reduced anti-tumor effects. To overcome these challenges, the development of a drug delivery system that delivers photosensitizers specifically to cancer cells has been attempted [9,10]; however, its clinical application has not yet been established.

Oncolytic viruses exhibit cancer-specific proliferation and cell-killing effects without affecting normal cells and can be classified into two types: naturally occurring and genetically modified [11]. Several viruses (such as herpes simplex virus, adenovirus, vaccinia virus, vesicular stomatitis virus, measles virus, and mammalian orthoreovirus (MRV) are currently used in oncolytic virotherapy [11,12,13,14,15,16,17]. Among them, MRV, which belongs to the family *Reoviridae*, is a naturally occurring oncolytic virus that is non-pathogenic in most adults [11]. MRV exclusively replicates in, and destroys, cancer cells where antiviral pathways cannot be activated. Clinical trials involving the use of MRV have been conducted for several cancers, such as glioma, melanoma, pancreatic, colorectal, lung, breast, and head and neck cancers [18,19,20,21,22,23,24]. We have also previously reported the antitumor effects of MRV in pancreatic, breast, and GC cell lines [16,25,26,27,28]. MRV is a non-enveloped virus with a genome consisting of ten segmented double-stranded RNAs (dsRNAs). Kobayashi et al. (2007) developed a plasmid-based reverse genetics system for MRV, in which 10 plasmids encoding each viral genomic cDNA were co-transfected into cells [29]. This system has enabled the generation of recombinant MRVs that express foreign genes [30,31]. Recently, we also generated a recombinant MRV expressing iRFP720 (a near-infrared fluorescent protein) for use in fluorescence-guided surgery and identified the genomic site that tolerates the functional expression of a foreign gene [32].

We believe that MRV could be a useful vector for sending photosensitizers to cancer cells. To this end, we selected the photosensitizer KillerRed (KR), a red fluorescent protein that produces ROS upon irradiation with green light [33]. This combination is expected to enable not only visualization but also photodynamic therapy for cancer cells in vivo. A similar concept has been reported using a recombinant adenovirus vector that can selectively replicate in cancer cells where telomerase is highly expressed [34,35]. Furthermore, although we previously showed that the recombinant MRV expressing iRFP720 had the low proliferation ability possibly due to the large size of the inserted gene [32], it was expected to be improved by the smaller size of the KR gene. In this study, to prove the concepts, we generated a recombinant MRV-expressing membrane-targeted KR (KRmem) and evaluated its cytoreductive effect after light irradiation on GC cells.

## 2. Results

### 2.1. Generation of a Recombinant MRV-Expressing KRmem

**Figure 1:**
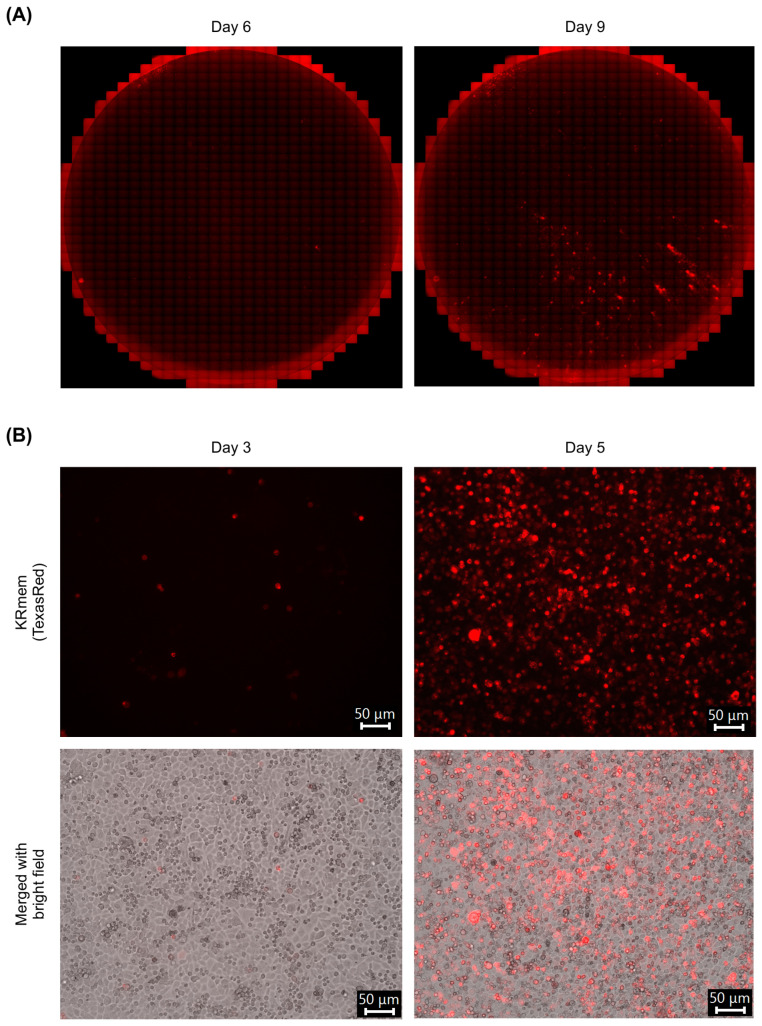
Recovery of the KRmem-expressing MRV. The BHK/T7-9 cells in a 6-well plate were transfected with 10 genome plasmids, including the recombinant S2 segment plasmid. Using the fluorescence microscope with the EVOS Light Cube for Texas Red, images were captured throughout a well and were combined using the tiling method. (**A**) The parts of the combined images at 6 and 9 days post transfection are shown. (**B**) The supernatant of the transfected cells was inoculated to BHK/T7-9 cells, and the fluorescence images of the KRmem expression were captured using the light cube for Texas Red. The fluorescent and merged images obtained at 3 and 5 days post infection are shown in pseudo-color.

To generate a recombinant MRV (strain T3D-L) expressing KRmem, BHK cells stably expressing T7 polymerase (BHK/T7-9 cells) were transfected with a plasmid encoding the recombinant S2 segment (pT7-T3D-L-S2/3′KRmem) together with nine plasmids encoding the other WT genome segments. In the pT7-T3D-L-S2/3′KRmem construct, the 2A peptide-KRmem was inserted in-frame into the 3′ end of the σ2-ORF. Six days post transfection, cells expressing fluorescence were observed, which subsequently spread to surrounding cells within nine days post transfection (Figure 1A). The supernatant containing the recombinant virus was collected and inoculated into fresh BHK/T7-9 cells in six-well plates. Three days after inoculation, we detected fluorescence-positive cells; by five days after inoculation, the fluorescence had spread throughout the well (as shown in Figure 1B). This outcome confirmed the successful passage of the recombinant virus, which was named T3D-L(S2/3’KRmem).

### 2.2. Characterization of the Recombinant MRV-Expressing KRmem

**Figure 2:**
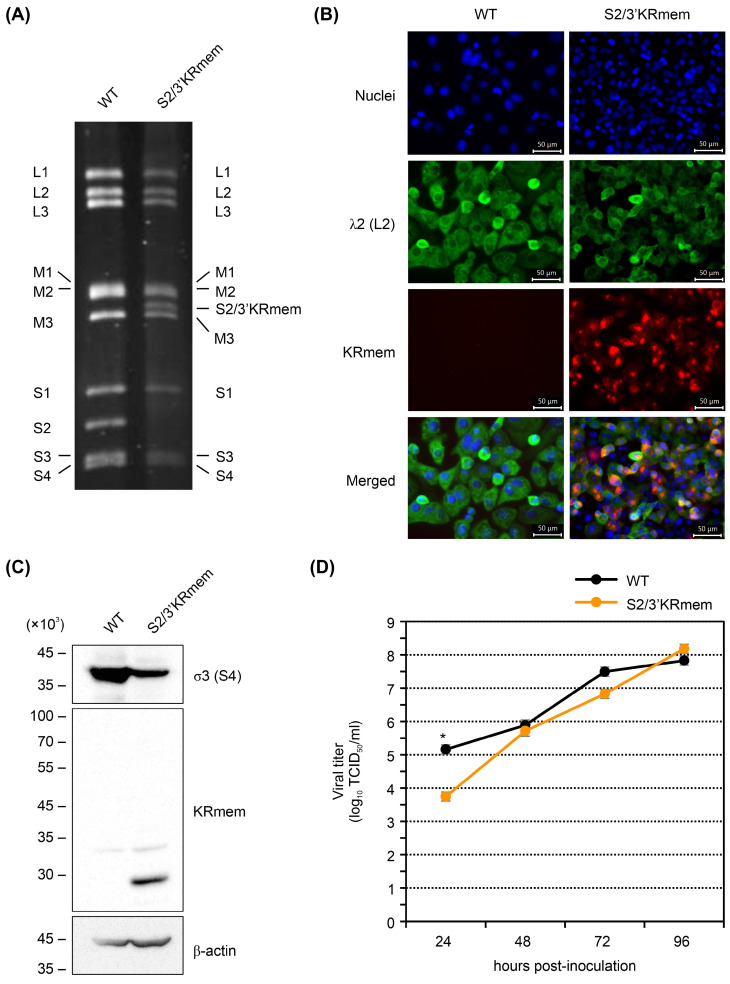
Properties of the KRmem-expressing MRV. (**A**) Electrophoretic patterns of genomic dsRNAs extracted from the wild type (WT) or KRmem-expressing virus, T3D-L(S2/3′KRmem). The name of each segment is also indicated. (**B**) Immunofluorescence assay of infected cells. Each virus was inoculated to BHK/T7-9 cells at a multiplicity of infection (MOI) of 100, and cells were fixed and stained with Hoechst 33342 (for nuclei) and the anti-λ2 (product of the L2 segment) antibody 2 days post inoculation. Images were obtained using the fluorescence microscope with the light cubes for DAPI (for nuclei), GFP (for the λ2 protein), and Texas Red (for KRmem). Merged images are also shown. (**C**) Western blot analysis of infected cells. Each virus was inoculated to BHK/T7-9 cells at an MOI of 100, and the cell lysate was collected 2 days post inoculation. Proteins in the lysate were separated by size, and the σ3 protein (product of the S4 segment), KRmem, and β-actin were probed using specific antibodies on the blotted membrane. Molecular weights are indicated on the left. (**D**) Growth curves of the WT and T3D-L(S2/3′KRmem) viruses in BHK/T7-9 cells. Each virus was inoculated to the cells at an MOI of 0.01, and viral titers in the culture supernatants harvested at the indicated time points were determined. Bars indicate standard deviation, and an asterisk shows the significant difference (*p* < 0.05, Student’s *t*-test, n = 3).

Electrophoresis of viral dsRNAs showed that the S2 segment of T3D-L(S2/3′KRmem) migrated between the M2 and M3 segments (Figure 2A), indicating that T3D-L(S2/3′KRmem) had a longer S2 segment than the WT virus. The immunofluorescence assay showed that KRmem expression was consistent with viral λ2 protein expression in T3D-L(S2/3′KRmem)-infected cells (Figure 2B). Western blot analysis revealed that the KRmem protein was detected at the expected size (258 aa), but not as a fusion protein with the viral σ2 protein, in T3D-L(S2/3′KRmem)-infected cells (Figure 2C). This indicated that the KRmem protein was separately translated from the fused gene by the T2A self-cleaving peptide. The growth activity of T3D-L(S2/3′KRmem) in BHK/T7-9 cells was comparable to that of the WT virus (Figure 2D).

### 2.3. Infection of Cell Lines with the Recombinant MRV-Expressing KRmem

**Figure 3:**
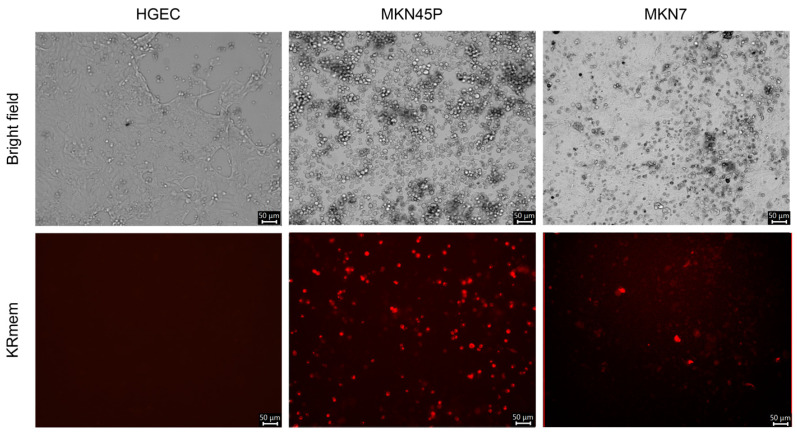
Infection of the KRmem-expressing MRV in gastric cancer cell lines. Normal human gastrointestinal epithelial cells (HGEC) and gastric cancer (GC) cell lines (MKN45P and MKN7) were inoculated with T3D-L(S2/3′KRmem) at 2000 TCID_50_/cell. The KRmem expression was observed using the fluorescence microscope with the light cube for Texas Red 7 (normal cells) or 5 (GC cell lines) days post inoculation, and captured fluorescent images are shown in pseudo-color.

We inoculated T3D-L(S2/3′KRmem) to normal human gastrointestinal epithelial cells (HGECs) at 2000 TCID_50_/cell; however, no fluorescence-positive cells were observed 7 days post inoculation (Figure 3). Conversely, fluorescence-positive cells were detected in two GC cell lines (MKN45P and MKN74) inoculated with T3D-L(S2/3′KRmem) at 5 days post inoculation. The percentages of KRmem-positive cells were approximately 35% and 10% for MKN45P and MKN7 cells, respectively (Figure 3).

### 2.4. Cytocidal Effect of Irradiation on MKN45P Cells Infected with the Recombinant MRV-Expressing KRmem

**Figure 4:**
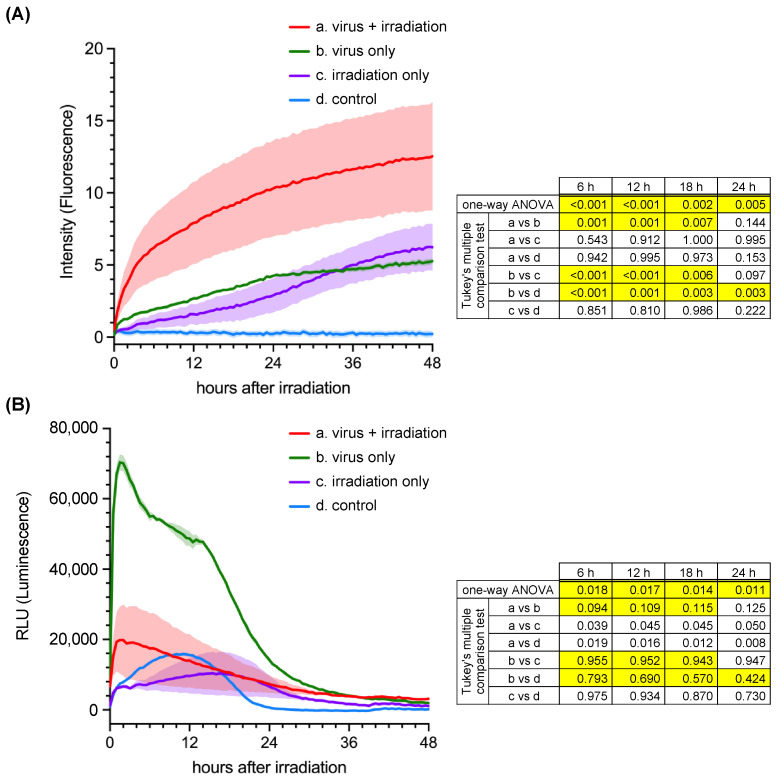
Photodynamic effect on MKN45P cells infected with the KRmem-expressing MRV. MKN45P cells were inoculated with/without T3D-L(S2/3′KRmem). Two days post inoculation, cells were irradiated with/without an orange laser (589 nm, 172 J/cm^2^). Afterwards, the cells were incubated in the medium containing the apoptosis/necrosis detection reagent, and (**A**) fluorescence for necrosis and (**B**) luminescence for apoptosis were measured every hour until 48 h after irradiation. Controls indicate cells without both virus infection and light irradiation. Error bands (shaded areas) show the standard error, and *p* values by one-way ANOVA and Tukey’s multiple comparison test (n = 4) are indicated for 6, 12, 18, and 24 h post irradiation in the right table. The significant differences (*p* < 0.05) were highlighted.

We evaluated the potential of the recombinant virus in PDT using MKN45P cells, which showed the highest susceptibility to the T3D-L(S2/3′KRmem) virus among the GC cell lines tested. The cytocidal effect was monitored in real time using the real-time Glo Annexin V Apoptosis and Necrosis Assay Kit. Necrosis was immediately detected at significantly higher levels in the virus-infected cells after light irradiation (Figure 4). In contrast, apoptosis was detected at significantly higher levels in virus-infected cells without light irradiation. However, the level of apoptosis in virus-infected cells with light irradiation was low, comparable to that in uninfected cells with or without light irradiation.

## 3. Discussion

In this study, we generated a recombinant MRV which expressed KRmem, named T3D-L(S2/3′KRmem). KRmem fused to the T2A sequence inserted into the 3′ end of the S2 segment was appropriately translated and functionally expressed in cells infected with recombinant MRV. Furthermore, the effect of insertion on viral replication in cultured cells was minimal. The self-cleaving 2A peptide is used to express foreign genes in segmented RNA viruses [30,36,37]. For MRV, as one genomic segment codes for one protein gene, the upstream (5′ end) or downstream (3′ end) of the viral gene can be the site for insertion of a gene cassette for bicistronic expression. The 5′ and 3′ end regions of each segment of MRV function as the packaging signal, which is a cis-acting element required for viral genomic RNAs to be packaged into virus particles. Additionally, the packaging signal sequence has been precisely determined in the L1, M1, and S2 segments [38,39,40]. Previously, we evaluated the possible insertion sites of the iRFP720 expression cassette for bicistronic expression and found that the 3′ end of the M1 segment was suitable for insertion [32]. Furthermore, recombinant MRV with the iRFP720 expression cassette inserted into the 3′ end of the S2 segment could be recovered; however, the insertion was genetically unstable [32]. In contrast, we generated recombinant MRV with the KRmem expression cassette inserted into the 3′end of the M1 segment, T3D-L(M1/3′KRmem), in the present study; however, it has not been evaluated further because it clearly showed lower growth activity than T3D-L(S2/3′KRmem). Unlike iRFP720 gene (951 bp) insertion [32], T3D-L(S2/3′KRmem) was genetically stable in the present study. This stability was likely attributed to the insertion of a smaller gene (774 bp), which had a smaller effect on viral replication activity. Thus, our findings suggest the 3′ end of the S2 segment as a possible candidate for the insertion of a foreign gene less than 774 bp in size.

Since robust KRmem expression was detected in T3D-L(S2/3′KRmem)-infected BHK/T7-9 cells, we evaluated the potential of the recombinant virus in photodynamic virotherapy using MKN45P (a GC cell line). To evaluate the cytocidal effect of the recombinant virus, we used a commercial kit that can detect early apoptosis and subsequent necrosis. The kit can measure the exposure of phosphatidylserine (PS) outside the lipid bilayer (cellular membrane) during apoptosis [41] using the PS-binding protein annexin V tagged with a split luciferase. It can also simultaneously detect membrane impairment using a cell-impermeant DNA dye, which indicates necrosis following apoptosis or other non-apoptotic cell death. MRV is known to induce apoptosis in infected cells [42]. This is consistent with our observation that apoptosis was predominantly detected in MKN45P cells infected with the recombinant virus, T3D-L(S2/3′KRmem), even without light irradiation. In contrast, necrosis was strongly detected in T3D-L(S2/3′KRmem)-infected cells after light irradiation, which is consistent with reports in the literature that KRmem could quickly cause cell death due to membrane lipid oxidation and compromised membrane integrity after light irradiation [43,44]. Consequently, the detection of apoptosis would be strongly hampered due to membrane disruption. Thus, the cytocidal effect of PDT using T3D-L(S2/3KRmem) was confirmed in GC cells in vitro.

In addition, necrosis was observed in non-infected cells after light irradiation but not in non-infected cells without irradiation. Irradiation of biological tissue for 5 min at 560 mW is mainly used as photochemical energy [45]. However, it may have been converted to thermal energy by substances contained in the culture medium, causing necrosis in vitro. Therefore, the irradiation method and energy density conditions must be set strictly for clinical applications.

T3D-L(S2/3′KRmem) could infect and replicate in GC cell lines but not in normal gastrointestinal epithelial cells. However, the infectivity in GC cell lines was not high, as shown previously [32]. Junctional adhesion molecule A (JAM-A) has been identified as an entry receptor for MRVs [46,47] and is downregulated in several cancer cells [48]. To confer JAM-A-independent infectivity to MRV, the attachment protein, σ1, encoded by the S1 segment needs to be modified. The introduction of an Arg-Gly-Asp (RGD) peptide into the σ1 protein has been shown to confer integrin αV-dependent infectivity on MRV, resulting in a modified virus causing JAM-A-independent cell lysis [49]. Moreover, the G196R mutation in σ1 was found to enhance sialic acid-dependent infectivity, and the mutant MRV was capable of JAM-A-independent infection [50]. Thus, these or other new modifications to expand MRV cell tropism will be the next step in the clinical application of photodynamic virotherapy using recombinant MRV. Alternatively, the treatment of a histone deacetylase inhibitor (HDACi) will be an option because Stiff et al. [51] showed that HDACi induced JAM-A expression and thereby facilitated MRV infection in multiple myeloma cells.

Although the delivery of the KR gene to target cancer cells in vivo is an issue in PDT using the photosensitizer KR protein, a viral vector can be used to overcome this challenge [43]. In this study, we demonstrated that recombinant MRV-expressing KRmem has the potential to deliver a photosensitizer specific to cancer cells, such as GC cells. Furthermore, because oncolytic virotherapy can also induce antitumor immunity [28,52], photodynamic virotherapy is expected to have better anti-tumor effects than PDT with a photosensitizer alone. The potential and usefulness of KR-expressing viruses in photodynamic virotherapy have already been reported with the adenovirus vector [34,35]; however, alternative recombinant viruses should be prepared to select the appropriate virus strain for each carcinoma and to respond to cases in which a patient has immunity against a recombinant virus used for therapy. We believe that recombinant MRV expressing KRmem may be an option. Furthermore, MRV can selectively infect and replicate in tumor cells in tumor xenograft mice after intravenous or intraperitoneal injection [16,21,30]; thus, it also has an advantage in terms of photosensitizer delivery. In the future, the cell tropism of recombinant MRV-expressing KRmem should be improved and validated using an animal model for the clinical application of photodynamic virotherapy in GCs. Furthermore, we believe that the improved recombinant MRV would be useful for the intraoperative diagnosis of micrometastases such as peritoneal dissemination.

## 4. Materials and Methods

### 4.1. Cells and Viruses

BHK/T7-9 cells [53], which stably express T7 RNA polymerase were provided by Dr. Ito N and Sugiyama M (Gifu University, Japan) and maintained in EMEM supplemented with 5% (*v*/*v*) fetal bovine serum (FBS), 10% (*v*/*v*) tryptose phosphate broth (TPB), and antibiotics. HGECs were obtained from Cell Applications Inc. (San Diego, CA, Canada). Two GC cell lines (MKN45P and MKN7) were obtained from the RIKEN BioResource Center (Ibaraki, Japan) and maintained in RPMI-1640 containing 10% FBS and antibiotics. The previously generated cDNA-derived MRV strain type 3 Dearing (rT3D-L) (32) was used as the wild type (WT) in this study.

### 4.2. Plasmids

**Figure 5:**
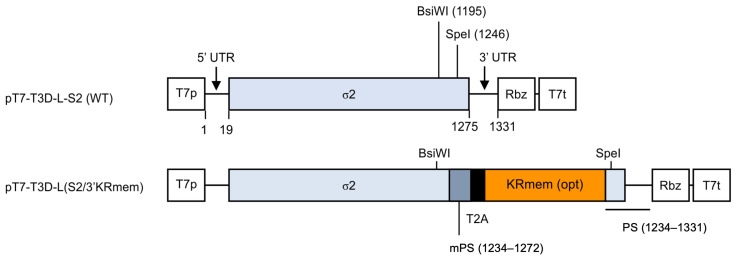
Schematic diagram of the recombinant viral genome in the genomic plasmid. The MRV S2 segment consists of the viral protein σ2 coding region and 5′ and 3′ untranslated regions (UTRs) at both ends. Its cDNA, together with the ribozyme (Rbz) sequence of the hepatitis D virus, were inserted between the T7 promoter (T7p) and terminator (T7t) sequences in the genome plasmid. The gene cassette consisting of the membrane-targeted and codon-optimized KillerRed gene [KRmem (opt), orange box] and the T2A peptide sequence (black box) were inserted into the 3′ end of the σ2 coding region (nt 19–1275, pale blue box) of the S2 segment using BsiWI and SpeI sites. The cassette was placed upstream of the packaging signal (PS, nt 1234–1331, underlined) located at the 3′ end of the segment. The sequence corresponding to the coding region in PS was not translated because the KRmem gene possessed a stop codon. Several silent mutations were introduced into the duplicated coding region corresponding to PS (mPS, nt 1234–1272, gray blue box) to prevent deletion of the cassette by homologous recombination, and the SpeI site in mPS was simultaneously abolished.

Ten previously described plasmids were used to rescue the MRV strain T3D-L (pT7-T3D-L-L1, -L2, -L3, -M1, -M2, -M3, -S1, -S2, -S3, and -S4) (29). Each cDNA of the viral genome segment was inserted between the T7 promoter and hepatitis delta virus ribozyme sequence. In this study, we constructed a modified genome plasmid of the S2 segment, pT7-T3D-L(S2/3′KRmem), to generate recombinant MRV-expressing KRmem (Figure 5). A gene cassette consisting of the codon-optimized KRmem joined to the T2A (a self-cleaving 2A peptide of *Thosea asigna* virus) sequence with parts of the viral S2 segment cDNA was artificially synthesized and purchased from Genewiz Japan (Tokyo, Japan).

### 4.3. Recovery of Recombinant MRV

The recovery of the recombinant MRV, T3D-L(S2/3′KRmem), was performed as previously described [32]. Briefly, 10 genome plasmids were co-transfected into BHK/T7-9 cells seeded in a 6-well plate using the TransIT-LT1 transfection reagent (Mirus Bio, Madison, WI, USA). Images of transfected cells were obtained using an EVOS FL Auto2 fluorescence microscope with EVOS Light Cube Texas Red (Thermo Fisher Scientific, Waltham, MA, USA). Fluorescence images covering the entire well were captured and combined into one tiled image using the operating software installed in the microscope system. Culture supernatant was collected 9 days post transfection and stored at −80 °C in small aliquots until use. Subsequently, the recovered virus was amplified in BHK/T7-9 cells, and the supernatant was concentrated via ultracentrifugation as follows: 20 mL of the collected supernatant was divided into two 9.5 mL tubes (70 mL, polycarbonate bottle; Beckman Coulter Inc., Brea, CA, USA). Ultracentrifugation was performed at 100,000× *g* for 1.5 h at 4 °C using an Optima L-80 XP ultracentrifuge with a 45Ti rotor (Beckman Coulter, Inc.). After ultracentrifugation, the supernatant was discarded and the pellet was dissolved in 200 μL of FBS-free medium. The amplified and concentrated virus solutions were aliquoted and stored at −80 °C until use.

### 4.4. Virus Titration

The viral titer, which is the median tissue culture infectious dose (TCID_50_), was determined in a 96-well plate as described previously [32]. Briefly, 10-fold serial dilutions of the viral solution and cell suspension (BHK/T7-9 cells) were added to each well. The plate was incubated at 37 °C for 7 days and observed daily for the presence or absence of cytopathic effects in the WT virus or fluorescent cells in the recombinant virus. Viral titers were calculated using the Spearman–Kärber method.

### 4.5. RNA Electrophoresis

BHK/T7-9 cells were infected with the WT or T3D-L(S2/3′KRmem) virus, and total RNA was purified from the supernatant and cells using TRIzol LS reagent (Thermo Fisher Scientific). The RNA sample was mixed with an equal volume of the sample buffer solution with 2-mercaptoethanol (2×) for sodium dodecyl sulfate-polyacrylamide gel electrophoresis (SDS-PAGE) (Nacalai Tesque, Kyoto, Japan) and electrophoresed on a 10% gel at 30 mA for 130 min in an ice bath. dsRNAs were visualized using SYBR Gold nucleic acid gel staining (Thermo Fisher Scientific).

### 4.6. Immunofluorescent Assay

The virus-infected BHK/T7-9 cells were fixed with 4% paraformaldehyde and permeabilized with 0.2% Triton X-100. Cells were incubated with an anti-λ2 monoclonal antibody (mAb) (clone 8F2, Developmental Studies Hybridoma Bank at the University of Iowa, Iowa, IA, USA) and stained with Alexa Fluor 488-conjugated anti-mouse IgG (H + L) cross-adsorbed secondary antibody (Thermo Fisher Scientific) and Hoechst 33342 solution. The stained cells were imaged using an EVOS FL Auto2 with a Light Cube for DAPI, GFP, and Texas Red.

### 4.7. Western Blot

The virus-infected BHK/T7-9 cells were lysed with lysis buffer (1% (*v*/*v*) NP-40) and mixed with an equal volume of the sample buffer for SDS-PAGE (Nacalai Tesque). The heat-denatured sample was subjected to SDS-PAGE in a 10% gel, and the separated proteins were electrotransferred to an Immobilon-P polyvinylidene difluoride (PVDF) membrane (Merck Millipore, Burlington, MA, USA). The membrane was blocked with phosphate-buffered saline containing 5% (*w*/*v*) skim milk and 0.1% (*v*/*v*) Tween-20, and it was incubated with mouse anti-σ3 mAb (clone 4F2, Developmental Studies Hybridoma Bank), mouse anti-β-actin mAb (clone G043, Applied Biological Materials, Vancouver, BC, Canada), or rabbit anti-KillerRed polyclonal antibody (Evrogen, Moscow, Russia). The membrane was then treated with SuperSignal West Femto Maximum Sensitivity Substrate (Thermo Fisher Scientific) and the chemiluminescent signal was detected using LuminoGraph I (ATTO, Tokyo, Japan).

### 4.8. Virus Growth in Cells

Multi-step viral growth in BHK/T7-9 cells was examined as described previously [32]. In brief, each virus was inoculated into monolayered cells at a multiplicity of infection (MOI) of 0.01, and the inoculated cells were incubated at 37 °C. The culture medium was harvested after 24, 48, 72, and 96 h of incubation, and viral titers were determined as described above.

### 4.9. Infection of HGECs with Recombinant MRV

The HGECs (2 × 10^5^ cells) and the T3D-L(S2/3′KRmem) virus (2000 TCID_50_/cell) were added to a well of a 24-well plate and incubated at 37 °C. The inoculated cells were observed daily, and fluorescence images were obtained as described above. The percentage of fluorescent-positive cells was analyzed using ImageJ [54].

### 4.10. Infection of the GC Cell Lines with Recombinant MRV

The GC cells (1 × 10^5^ cells) and the T3D-L(S2/3′KRmem) virus (2000 TCID_50_/cell) were added to a well of a 24-well plate and incubated at 37 °C. The inoculated cells were observed daily, and fluorescence images were obtained as described above. The percentage of fluorescent-positive cells was analyzed using ImageJ (version 1.54f) [54].

### 4.11. Photodynamic Effect on the Recombinant MRV-Infected GC Cell Line

MKN45P cells (3 × 10^5^ cells) and the T3D-L(S2/3′KRmem) virus (100 TCID_50_/cell) were added to the wells of a 96-well plate and incubated at 37 °C. Two days post inoculation, the culture media of infected cells were replaced with phenol red-free RPMI medium containing 10% FBS. Subsequently, the cells were irradiated using an orange laser (589 nm) system (Sanz, Tokyo, Japan) at 560 mW for 5 min (168 J/cm^2^). The cells were then incubated in a medium containing the detection reagent of the Real-time Glo Annexin V Apoptosis and Necrosis Assay kit (Promega, Madison, WI, USA), and the fluorescent and luminescent signals were measured every hour (until 48 h after irradiation) using a Varioskan LUX multimode microplate reader (Thermo Fisher Scientific).

### 4.12. Statistical Analysis

Data were statistically evaluated using the t-test or the one-way ANOVA with Tukey’s multiple comparison test in SPSS software (version 25.0; IBM Corp., Armonk, NY, USA) or EZR (version 4.03; Saitama Medical Center, Jichi Medical University, Saitama, Japan) [55], respectively. More precisely, it is a modified version of R commander (version 2.7-1) that was designed to add statistical functions frequently used in biostatistics. Statistical significance was set at *p* < 0.05.

### 4.13. Image Processing

Fluorescence images were obtained using a high-sensitivity interline CCD monochrome (grayscale) camera in the EVOS FL Auto2. Pseudo-colors were applied to the captured grayscale images using ImageJ; brightness and contrast were adjusted.

## 5. Conclusions

Based on the oncolytic MRV, we have generated a new recombinant virus expressing the KRmem gene in this study. This recombinant virus was able to induce necrosis in the GC cell line by photo irradiation but not able to replicate in normal gastrointestinal epithelial cells. Hence, we provide a proof of concept that MRV-expressing KRmem could serve for the combination of cancer visualization and photodynamic therapy, which would be a novel treatment tool for GC.

## Data Availability

All data generated or analyzed during this study are included in this published article.
